# Factors associated with experimentation of electronic cigarettes among Parisian teenagers in 2013

**DOI:** 10.1186/s12971-015-0065-4

**Published:** 2015-12-16

**Authors:** Bertrand Dautzenberg, Ivan Berlin, Marie-Laure Tanguy, Nicole Rieu, Pierre Birkui

**Affiliations:** Paris Sans Tabac (PST), Paris, France; University Hospital Pitié Salpêtrière-Charles Foix (APHP), Paris, France; Université Pierre et Marie Curie (upmc), Faculté de médecine, Paris, France; Rectorat Académie de Paris, Paris, France; Service de pneumologie – Hôpitaux universitaires Pitié Salpêtrière-Charles Foix, 91 Bd de l’Hôpital, 75651 Paris Cedex 1, France

**Keywords:** Electronic cigarette, Teenagers, Survey

## Abstract

**Background:**

Electronic cigarettes (e-cig.) became widely used among adults. Data are insufficient about e-cig. experimentation among youth.

**Methods:**

To assess prevalence of e-cig. experimentation and associated factors among the 12 to 19 years old we analyse a cross sectional school based survey in the city of Paris, France in 2013 on a randomly selected sample of 2 % of schoolchildren (*n* = 3 279). Self-report questionnaire include demographic, individual and family smoking characteristics and questions about e-cig: *“Have you ever used an e-cigarette?”-* “*Did you use e-cigarette in the last 30 days?*”-“*Did you try e-cigarette as a first tobacco product?*”

**Results:**

In 2013, 17.9 % (564) schoolchildren reported having experienced the e-cig (boys: 19.0 %, girls: 16.8 %) compared to 9.8 % in 2012. Experimentation rate increases from 5 % among the 12 to 30 % among the 16-year-old. E-cig. experimentation was significantly associated with 11 parameters including : age >15 years (OR: 0.66 (IC95 % = 0.46–0.94)); smoking 10 cigarettes or more (OR = 5.67 (IC95 % = 3.11–10.34)), best friends and siblings smoker (OR = 1.54 (IC95 % = 1.11–2.14)) and (OR = 1.88 (IC95 % = 1.41–2.52)); experimentation of shisha (OR = 2.60 (IC95 % = 1.75–3.86)), cannabis use (OR = 1.90 (IC95 % = 1.32–2.72)); having two parents who forbid smoking (OR = 2.32 (IC95 % = 1.63–3.30)). Only 5.6 % of the study population (and 32.5 % of e-cig. experimenters (183/564)) have used it in the last 30 days; 1.7 % of the study population and 10.0 % of e-cig. experimenters were non-smokers (56/564).

**Conclusions:**

Rate of e-cig. experimentation among schoolchildren increased by 8.1 % in 1 year. Non-smoking youth may use e-cig. Prospective studies are urgently needed to assess the evolution of e-cig. use both among smoking and non-smoking youth.

## Background

Electronic cigarettes (e-cig) are a new product that has reached some maturity; currently available devices seem to be more secure than those available several years ago [1]. E-cig. appear to be less toxic than conventional cigarettes if used by a smoker [2, 3]. Only some public health advisors and physicians are neutral, some argue for a prohibition or severe restriction of the e-cig. use [4, 5] when others encourage adult smokers to switch from tobacco to e-cig. [6, 7], but all agree to keep as low as possible the use of e-cig by teenagers [8].

E-cig. are widely considered as a product that may help smokers quit, but, as of today, there is only little evidence supporting this [9]. Moreover, there is a concern that long term use may induce initiation of conventional cigarette or other types of tobacco use among youth. Although the long-term effect of e-cig’ use among youth can only be assessed by prospective studies, there is an urgent need to generate knowledge even by cross-sectional studies about factors associated with its use to accumulate as quickly as possible new data.

Paris Sans Tabac (PST), a non-profit foundation aiming to reduce prevalence of tobacco in Paris, conducts yearly surveys of schoolchildren aged 12 to 19 years. A question about e-cig. experimentation was introduced in 2012 [10] and in the 2013 survey two more questions have been added. We report here about factors associated with the experimentation of e-cig. among Parisian schoolchildren.

## Methods

### The PST survey

From 1991 on a cross-sectional anonymised survey has been conducted every year among 2 % of schoolchildren randomly selected by class clusters from the list of classes (4 842 public and 2 424 private) provided by Paris’ school authorities with quotas by class level from both private and public schools to provide a representative sample of the 188 000 schoolchildren of the City of Paris.

The one page self-report questionnaire is distributed and collected with the help and explicit agreement of school authorities of the City of Paris. Every year, one of the usual teachers, with or without the help of the school nurse, distributes and collects the questionnaire in the designated classes. In 2012 the following question was added to the standard questions: “*Have you ever used an e-cigarette?*” In 2013 two further questions were added: “*Did you use e-cigarette in the last 30 days?*” and “*Did you try e-cigarette as a first tobacco product?*”

The standard questionnaire contained the following items: age, gender, smoking status (smoker, former smoker, non-smoker) of the father, mother, brothers, sisters and best friend; prohibition of tobacco use by one or two parents; ever use of tobacco; age of the first cigarette smoked, current smoking status (daily smoker, occasional smoker, former smoker, non-smoker); experimentation with shisha (hookah) and with cannabis; amount of use of alcohol last month; intention to quit tobacco. The classes’ identification provides information about studying in a private or a public school which allows approximating by the City’s website the mean income/inhabitant of the school district. Upon completion, the questionnaires are scanned and analysed with the Neoptec® software (Marseille, France).

### Missing data’s

A total of 3 279 questionnaires, who have no missing data on age and smoking status was are included in the study. The missing date are less 5 % for most of questions: For the question on experimentation of e-cig 3.9 % of answers are missing (*n* = 128). For the multivariate analysis 31.5 % of questionnaires had at least a missing value for one of the 9 parameters included in the analysis, so the multivariate analysis has been performed on 69.5 % of the whole population (2 279).

### Data analysis

Data are reported as frequencies (percent) and number. Potential associations with experimentation of e-cig. were studied using chi-square tests. Variables that were significant in univariate analyses at *p* ≤ 0.05 were included in a stepwise logistic regression model. The alpha level was set at 0.05.

All analyses were performed with the SAS software version 8.2 (SAS Institute®, Cary, NC).

## Results

### Study population

A total of 3 279 schoolchildren completed the questionnaire. Their age ranged from 12 to 19 years. Forty eight percent of the sample was male. In this population the rate of non-smokers is 72.4 %, of daily or occasional smokers is 26.1 % and the rate of former smokers 1.6 %.

### Experimentation of electronic cigarette

**Fig. 1:**
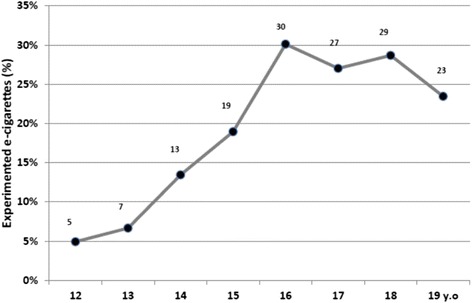
Electronic cigarette experimentation rate by age in 2013 among 12–19 years old Paris schoolchildren

In 2013, 564 schoolchildren reported having experimented at least once the e-cig., 2 587 had not and 128 answers were missing. The rate of e-cig. experimenters is 17.9 % among the 3 151 respondents (17.2 % of the whole population). The e-cig. experimentation rate was 16.8 % among girls and 19.0 % among boys. The e-cig. experimentation rate starts to increase among the 13 years old and reaches 30 % among the 16 years old (Fig. 1). The rate of experimentation of e-cig. is 7.1 % in non-smokers, 37.3 % in former smokers, 38.7 % in occasional smokers and 63.0 % in daily smokers.

### Factors associated with experimentation of electronic cigarettes

In univariate analyses the following variables were significantly associated with e-cig. experimentation and were included in the multivariate analysis: age, gender, smoking status (smoker, former smoker, non-smoker) of the father, mother, brother, sister and best friend; prohibition of tobacco use by one or two parents; ever use of tobacco, current smoking status (smoker, former smoker, non-smoker), experimentation of shisha, cannabis; use of alcohol in the last month, experience of binge drinking; studying in a private or a public school, mean income per inhabitant of the school area (Table 1). Two variables: intention to quit tobacco and age of the first cigarette were not included into the multivariate analysis because of no significant association with e-cig. experimentation.

**Table 1:** Mains factors associated to experimentation of electronic cigarette in the univariate analysis of the 2013 adolescent study in Paris

Effect	*n*	Non experimentation e-cig-(%)	Experimentation e-cig. (%)	*p*
Gender = male	3110	47.0 %	51.0 %	0.11
Age 12–14 years	3151	48.9 %	21.3 %	<0.0001
Ager 15–17 years		29.2 %	44.7 %	
Age > 17 years		21.9 %	34.0 %	
Ever cigarettes smoker	3078	31.8 %	91.0 %	<0.0001
Smoking status: non-smoker	3081	84.9 %	28.1 %	<0.0001
Smoking status: former smoker		1.1 %	3.2 %	
Smoking status: occasional smoker		8.9 %	27.1 %	
Smoking status: daily smoker		5.1 %	41.%	
Ever shisha use	3126	27.7 %	81.7 %	<0.0001
Father smoke	3055	27.7 %	35.%	<0.0001
Mother smoke	3099	20.3 %	31.7 %	<0.0001
Brothers and sisters smoke	2929	19.0 %	45.7 %	<0.0001
Best friend smoke	2989	17.2 %	57.9 %	<0.0001
Non agreement of parents on the prohibition to smoke	3048	6.0 %	11.7 %	<0.0001
Any alcohol las month	3070	37.3 %	78.5 %	<0.0001
More 4 glasses of alcohol in a day 4 times or more	2341	16.1 %	47.2 %	<0.0001
Cannabis experimentation	2891	88.7 %	41.8 %	<0.0001
Private school	3153	34.8 %	47.3 %	<0.0001
Mean income per inhabitant in the school area >49 K€	3151	22.8 %	31.9 %	<0.0001

### Multivariate analysis

The multivariate analysis was run with 2 279 questionnaires (69.5 % of the initial sample) because of missing data of at least one variable included in the model.

The higher determinants of e-cig. experimentation use were smoking 10 cigarettes or more (OR = 5.67 (IC95 % = 3.11–10.34)) and ever smoked a cigarette OR = 4.46 (IC95 % = 2.81–7.09)) but the likelihood of electronic cigarette experimentation is also associated with an age less than 15 years, with the current smoking status of the best friend and sibling; with the use of conventional cigarettes, shisha and cannabis, with the prohibition to smoke by at least one of the parents and being in a private school (Table 2).

**Table 2:** Factors associated to experimentation of electronic cigarette in the multivariate analysis in a 2013 adolescent survey Paris

Effect	O.R	95 % Wald confidence limits		*P* value
Age (>15 vs ≤15^a^)	0.66	0.46	0.94	0.0217
Smoking status of best friend (smoker versus non-smoker or ex-smoker^a^)	1.54	1.11	2.14	0.0089
Smoking status brother (smoker versus non-smoker or ex-smoker or only child^a^)	1.88	1.41	2.52	<.0001
Ever smoked a cigarette versus no^a^	4.46	2.81	7.09	<.0001
Ever smoked a shisha versus no^a^	2.60	1.75	3.86	<.0001
Cannabis use versus no^a^	1.90	1.32	2.72	0.0005
Smoking status:				
- No smoking^a^				
- Smoking less than 10 cigarettes	2.28	1.57	3.29	<.0001
- Smoking 10 cigarettes or more	5.67	3.11	10.34	<.0001
Private school (vs. public school^a^)	0.66	0.50	0.87	0.0035
Parental message of prohibition to smoke:				
- No prohibition^a^				
- Prohibition of one parent	1.84	1.08	3.12	0.0243
- Prohibition of two	2.32	1.63	3.30	<.0001

*OR* odds ratio

^a^ = Reference

### Non-smokers who experiment electronic cigarette

Among the non-smoking schoolchildren, 2.3 % of non-smokers and 9.9 % of e-cig. experimenters reported having experimented e-cig. (56/2 374). A total of 1.7 % of the study population were non-smokers and e-cig. experimenters (56/564). Among non-smokers who experiment e-cig. 36/56 were male and 45/56 had at least one parent prohibit to smoke. Non-smoker e-cig. experimenters were younger (mean: 15.5 years) than smokers who had experimented e-cig (mean = 17.1 years).

### Electronic cigarette as the first product experimented

Data on first experimentation of tobacco or tobacco related products were available for 383 schoolchildren. Twelve children (3.1 %) reported e-cig. as the first product used; 64.8 % (248) started smoking by standard cigarettes, 7.3 % (28) by roll your own tobacco, 8.9 % (34) by perfumed cigarette; 12.8 % (49) by using shisha and 3.1 % (12) reported having started to smoke by cigars or other tobacco products.

### Use of electronic cigarette in the last 30 days among experimenters of electronic cigarette

Of the 564 schoolchildren who reported having experimented e-cig., 128 provided no answer on recent use but 183/564 experimenters used it during the last 30 days (5.6 % of the study population).

A total of 114/128 experimenter schoolchildren who provided no answer on recent use of tobacco or tobacco related products are non-smokers of occasional smokers (Table 3).

**Table 3:** Use electronic cigarette during the last 30 days among the 564 experimenters of electronic cigarette according to their smoking status

	E-cig. use in the last 30 days	Non-responder last 30 days	No e-cig. use in the last 30 days	Percent of responders using e-cig. last 30 days^a^	Percent of the total sample using e-cig. last 30 days^a^
Non-smoker	38	87	32	54.3 %	24.2 %
Occasional smoker	53	27	76	41.1 %	34.0 %
Ex-smoker	5	9	4	55.6 %	27.8 %
Daily smoker	87	5	141	38.2 %	37.3 %
Total	183	128	253	42.0 %	32.4 %

^a^The rate of experimenter who had used electronic cigarette during last 30 days is presented as a rate of respondent and of the total study population

## Discussion

The survey identified several factors significantly associated with e-cig. experimentation among Parisian schoolchildren aged 12 to 19 years. E-cig. experimentation was associated with older age, smoking of a best friend or a brother, smoking of conventional cigarettes or shisha, using cannabis and being in a private school. Greater number of current cigarette smoking was dose-dependently associated with the likelihood of e-cig. experimentation. Interestingly, and paradoxically, prohibition of smoking by parents was associated with increased likelihood of e-cig. experimentation.

Among those who have experimented e-cig. only 1/3 use it the last 30 days. In the non-smoker sub group who tried e-cig. only 1/5 use it the last 30 days, more the question used in the study on e-cig. use during the last 30 days overestimated the number of daily users, non-assessed in this study.

The fact that near 10 % of the e-cig. experimenters are non-smokers may be an alert but the present data does not allow to conclude that the e-cig. is a gateway to smoking because of the cross-sectional nature of the survey.

### Strength and limitations

Strengths include the random selection of school classes, the relatively large sample size. Limitations include the cross-sectional nature of the study and the reduced representativeness for the whole sample of the multivariate analysis due to missing data. The findings cannot be generalizable to youth of same age in other geographical areas of France, or to other countries or regions than the present sample. As the study is a cross sectional study, association can indicate link between the studied parameters but not causality.

### Comparison to the 2012 survey in Parisian schoolchildren

The 2013 data can be compared historically to the 2012 data [10] because the questions were identical with the exception of those about e-cig. use. In February-March 2012, the e-cig. was a more confidential product which can explain why one of six Parisian schoolchildren, (16.9 %) (575/3409) did not provide answer to the question about e-cig. The response rate to the questions on e-cig. dramatically increased between 2012 and 2013: 96.1 % of the schoolchildren provided an answer on e-cig. in February-March 2013, probably because their increased awareness about the product. Similarly, the reported rate of e-cig. experimentation increased respectively from 8.1 % in 2012 to 17.2 % in 2013 of the total sample and from 9.8 % to 17.9 %, among the respondents.

### Comparison with others studies

Data about the e-cig. experimentation rate among youth are, as of today, scanty. The Eurobarometer 2012 [11] reports experimentation rate of 5 % in France, 6 % in Europe as of May 2012 among adults; it does not report on e-cig. experimentation rate among youth in France.

In 2010 a Korean survey reported an experimentation rate as low as 0.5 % among students [12]. In 2013 Dutra et al. *from the US student survey* report an experimentation of e-cig. of 3.1 % in 2011 and 6.5 % in 2012 [13]. In 2011, Goniewicz et al. [14] reported an experimentation rate in Poland of 23.5 % among the 15–19-year-old teenager population and 19.0 % among the 20–24-year-old students.

The 2012 Paris report found an experimenter rate of 8.1 % [10] close to the US report’s rate of the same year (6.5 %) [12]. The Paris 2013 survey [10] the rate of experimentation of 17.9 % is close to the rate of experimentation more than 2 years before (2010–2011) observed among Polish adolescents (23.8 %). As in the US student survey 2011–2012 [13], we observed a doubling of experimentation rate between 2012 and 2013.

### Use of electronic cigarettes in the last 30 days among experimenters

We found in the present study that the last 30 days e-cig. use among e-cig. experimenters is 32.4 % (42.0 % of responders) similar to that of the Polish study (34.9 %) [14] among the 15–19 years old. Two thirds of e-cig. experimenters do not use it in the last 30 days. Four studies [15–18] concerning adults report a lower rate than reported by our sample with 28–32 % conversion from experimentation to regular (or last 30 days) use. The Eurobarometer 2012 [11] reports a last 30-days use of e-cig. among experimenters of 20 % for France and 17 % for all Europe. According to these data 1/3 to 1/5 of adult e-cig. experimenters had used it during the last 30 days.

### Use of electronic cigarette by non-smokers

In the present study we identified 56 non-smokers who experimented e-cig. (9.9 % of e-cig. experimenters and 1.7 % of the study population). These non-smokers were younger than those young smokers who were e-cig. experimenters. Previous studies report only on very small proportions of non-smoker e-cig. experimenters: 1.6 % in a 2011 and 4.4 % in 2012 in the US study [13], 0.1 % in a Korean study [12], 1.4 % among Polish girls, 3.2 % among Polish boys of 15 to 19 years old (data of 2011) [14]. Our data shows higher non-smokers e-cig. experimentation rate.

This suggests that we urgently need prospective studies to learn whether and to what extent non-smoker e-cig. experimenters become conventional cigarette smokers, chronic e-cig. users or mixed users to elaborate more on place of e-cigarette in promotion or prevention of tobacco use in adolescents.

## Conclusions

E-cigarettes are mainly experiment by schoolchildren who had experienced regular cigarettes, had best friends and sibling smoker. A high tobacco consumption and the prohibition to smoke from the 2 parents is associated to an increased chance to use e-cigarette. Rate of e-cig. experimentation among schoolchildren increased by 8.1 % in 1 year from 2012 to 2013. Less 10 % non-smoking youth may use e-cig.

Prospective studies are urgently needed to assess the evolution of e-cig. use both among smoking and non-smoking youth.
